# Constipation Misperception: Could It Be Familial Adenomatosis Polyposis?

**DOI:** 10.7759/cureus.18656

**Published:** 2021-10-11

**Authors:** Priyanka Bhandari, Amit Sapra, Lauri Lopp, Christine E Albers, Sarah Hutchings

**Affiliations:** 1 Family and Community Medicine, Southern Illinois University School of Medicine, Springfield, USA

**Keywords:** familial adenomatosis polyposis, colon cancer, constipation, family history of colon cancer, hereditary cancer, early-onset colorectal cancer, apc gene, fap, tubular adenoma, colonoscopy

## Abstract

Colorectal cancer is the second leading cause of cancer deaths in the United States. Familial adenomatosis polyposis (FAP) is a rare cause of colorectal cancer. The United States Preventive Services Task Force (USPSTF) recommends screening for colorectal cancer in average-risk, asymptomatic adults aged 50 to 75 years. While age is the most important risk factor, we need to consider the family history of colorectal cancer. FAP is a rare cause of colorectal cancer, leading to high morbidity and mortality if undetected and undiagnosed. It is easy to overlook the family history in a busy primary care clinic with limited patient encounter times. Clinicians mustn't forget this important piece of information as it can give leads for further patient evaluation. We present a case report of a 21-year-old male who presented to our clinic to establish primary care and with vague abdominal complaints. Still, the concerning family history of early onset colon cancer in his half-sister raised red flags and directed us to further evaluate. Further evaluation revealed our patient, in fact, had FAP.

## Introduction

Familial adenomatous polyposis (FAP) is a hereditary form of colon cancer with an autosomal dominant mode of genetic transmission and an incidence of about 1 in 100000 newborns [1]. It is characterized by hundreds of polyps in the colon associated most commonly to a mutation in the Adenomatous Polyposis Coli gene located on 5q21 [1]. Most FAP patients have a family history of colorectal polyps and cancer, but around 30% of them can arise "de novo." The clinical presentation can vary from being completely asymptomatic to presenting with overt colonic and extra-colonic symptomatology [1]. The patients start developing polyps by the second and third decades of their life. If left untreated, there is a 100% risk of developing early onset colorectal cancer. The certainty of developing this cancer also implies high mortality of this young population from the same. Hence, it depends largely on the physician's suspicion and awareness. The patient might be asymptomatic or present with vague symptoms, but we may find other family members affected by taking a family history.

## Case presentation

**Figure 1:**
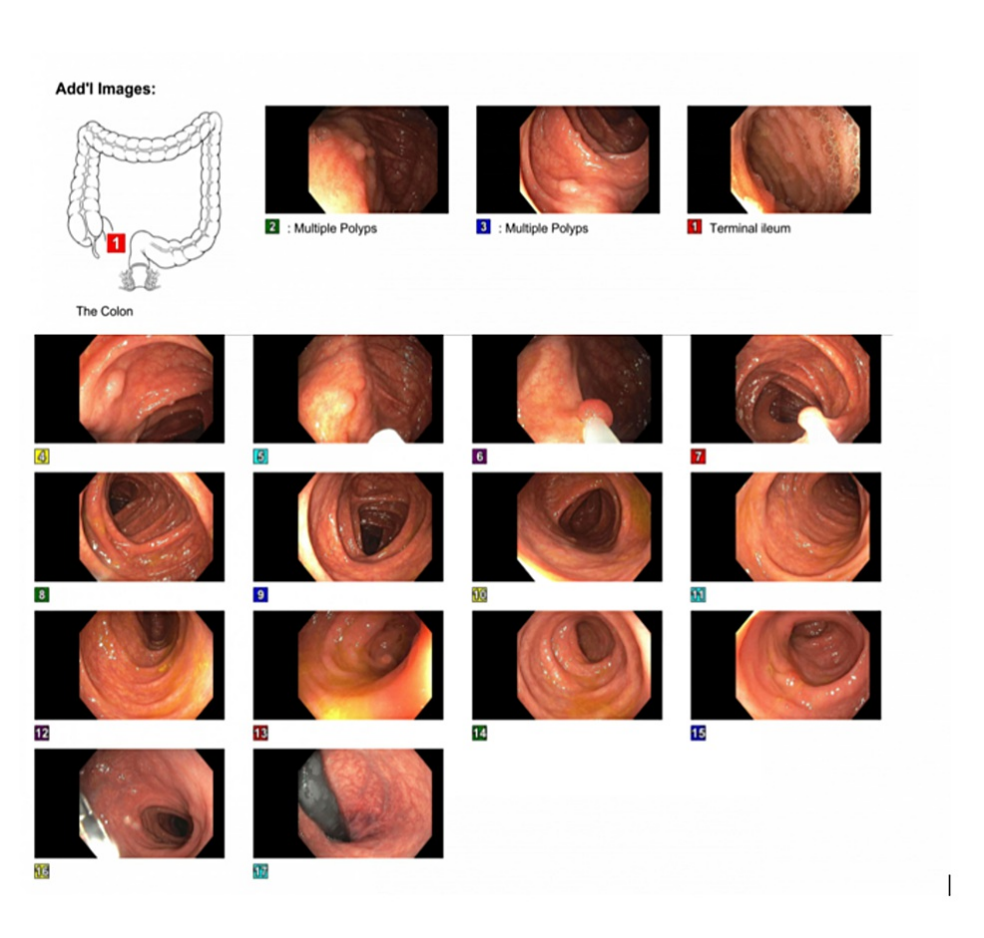
Colonoscopy showing multiple polyps

A 21-year-old male of mixed (African-American and Native American) race, with no known significant past medical history and currently not on any chronic prescription medications, presented to our family medicine clinic in December 2019 to establish primary care. The patient was not a good historian and was accompanied by his girlfriend in the clinic, who helped us corroborate history. His main concerns at the time of establishing care were low and anxious mood and intermittent constipation. The patient gave a history of constipation along with dull lower abdominal pain. The patient's stressors at home worsened his constipation, and the over-the-counter stool softeners and fibers only provided partial relief. He also gave a history of intermittent, painless, bright red bleeding per rectum. There was no history suggestive of fever, chills or rigors, nausea, vomiting, tenesmus, change in caliber of stools, hematemesis, melena, anorexia, or weight loss. His review of systems was unremarkable except described above. Family history was consistent with his half-sister being diagnosed with colon cancer in her early 20s, but history for other family members was unavailable at that time. Given the patient's current symptomatology and his positive family history of colon cancer, we decided about getting a diagnostic colonoscopy. He underwent the diagnostic colonoscopy in January 2020, which showed multiple sessile polyps in the entire colon (Figure 1).

**Figure 2:**
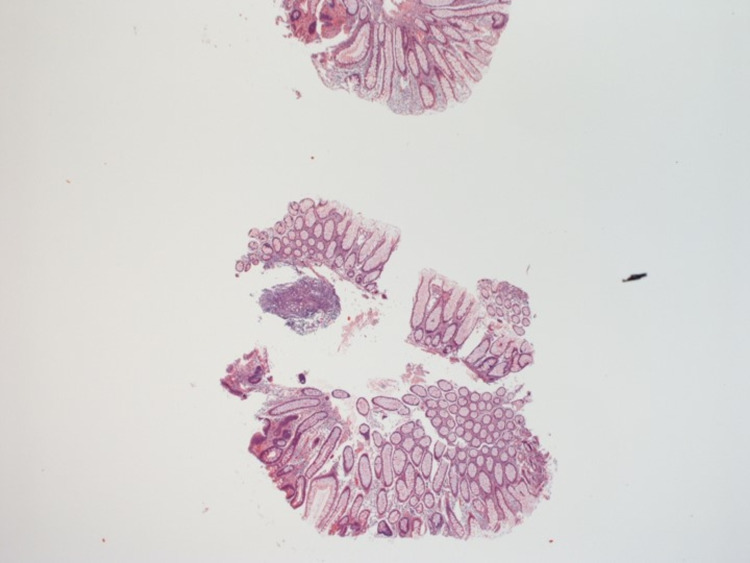
Histopathology of tubular adenoma from ascending colon (magnification: 20X)

**Figure 3:**
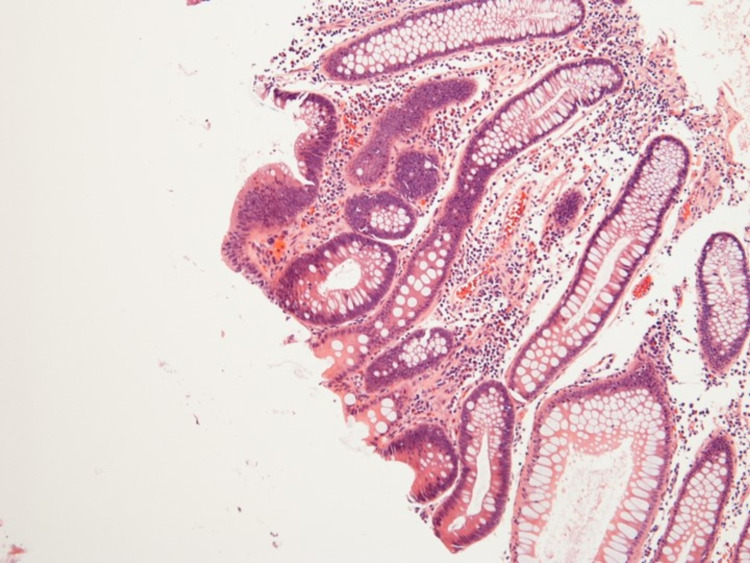
Histopathology of tubular adenoma from the ascending colon (magnification: 100X)

**Figure 4:**
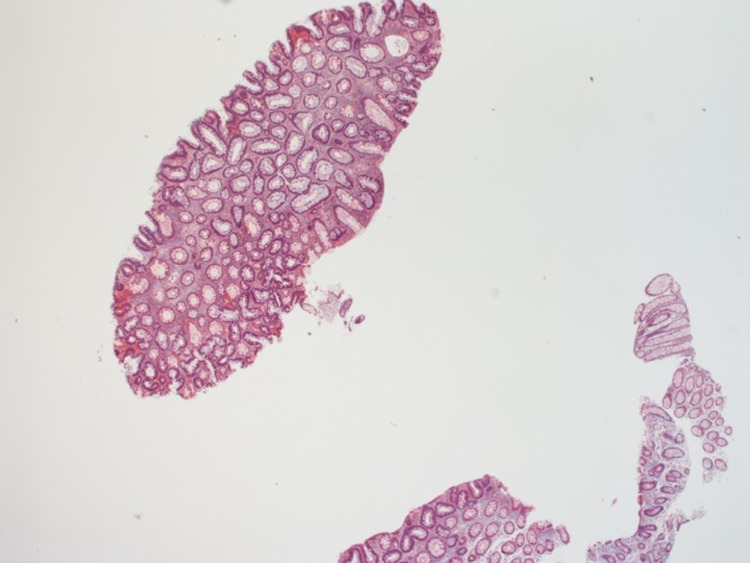
Histopathology of the tubular adenoma removed from the caecum (magnification: 20X)

**Figure 5:**
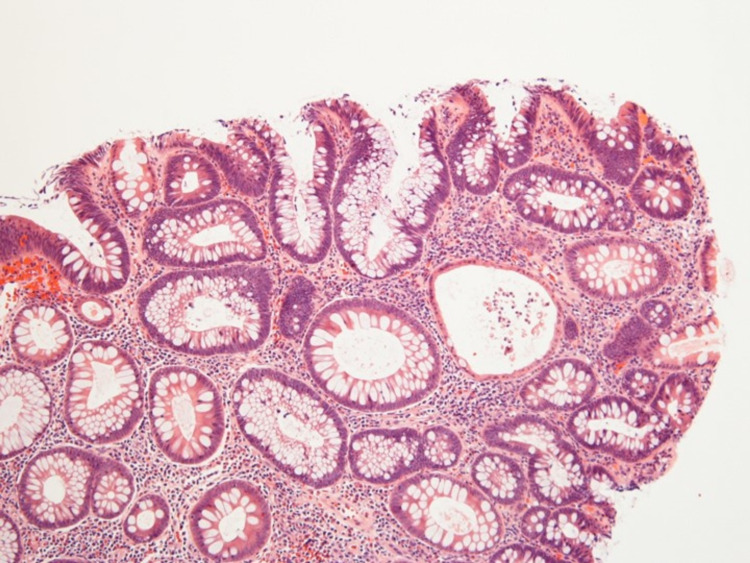
Histopathology of the tubular adenoma from the caecum (magnification: 100X)

These polyps were 2 to 10 mm in size. There were two large 10 mm polyps in the caecum and another two 10 mm polyps in the ascending colon, excised and determined to be tubular adenomas on histopathology (Figures 2-5).

The patient was referred immediately to the geneticist, who obtained a three-generation family history and constructed a pedigree. There was no evidence of consanguinity or Ashkenazi Jewish ancestry in the family. The patient had no full siblings but had "many" paternal half-siblings but was only familiar with two sisters. One of these sisters was healthy and was in her 30s, while the other had colon cancer with colon polyposis in her 20s. The patient was unfamiliar with his paternal family but believed that his father, a paternal aunt, a paternal uncle, and paternal grandmother all had colon/polyp issues. Other than his maternal grandmother with ovarian cancer at the age of 54 years, there was no maternal family history of cancer.

**Figure 6:**
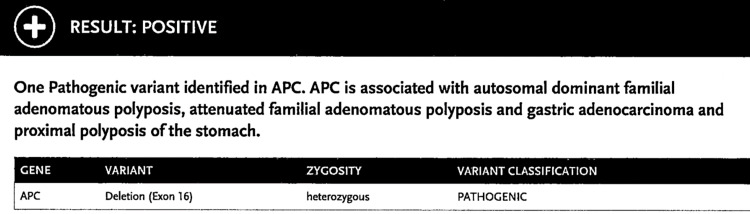
Genetic testing result of the patient

After collecting the family details, a history discussion was undertaken with the patient about the relationship between polyps and colorectal cancer. A presumptive diagnosis of FAP was made. As per the National Comprehensive Cancer Network (NCCN) Guidelines, the patient was also recommended an upper GI endoscopy, thyroid scan, serum testing, and therapeutic colectomy. After the patient agreed to the genetic testing, his blood sample was sent for analysis, which came back positive for a pathogenic partial gene deletion nine involving exon 16 consistent with a diagnosis of Classical FAP (Figure 6). He was also informed that all his biological children would have a 50% chance of inheriting FAP. He was also informed that he could go for in-vitro fertilization and use donor sperms for future pregnancies.

 

**Figure 7:**
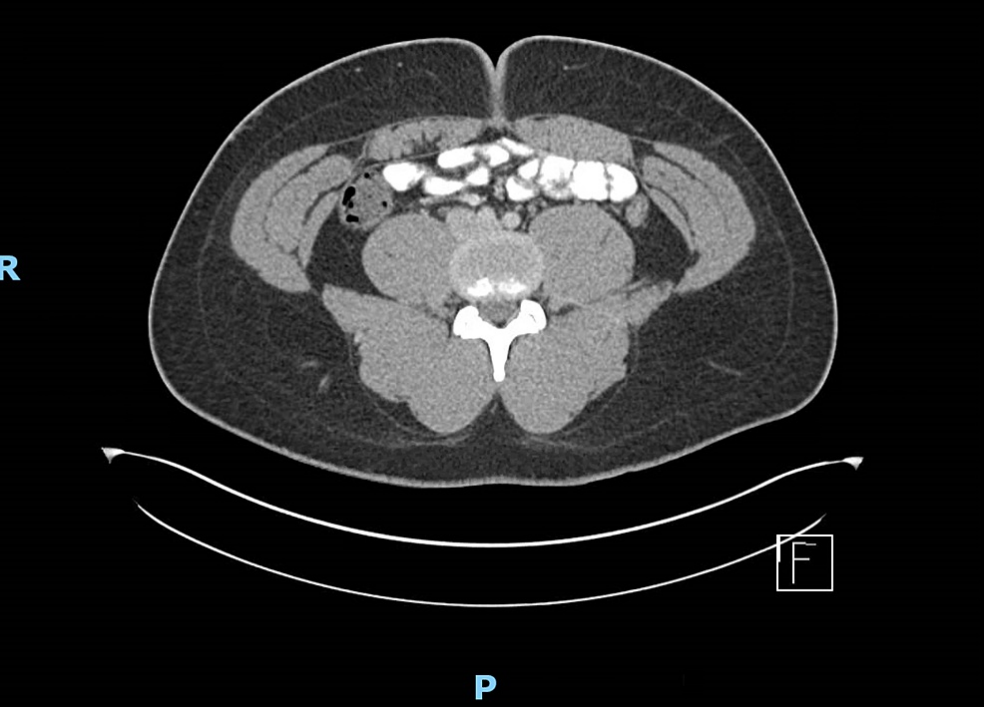
Computed tomography of the abdomen and pelvis did not show evidence of any extracolonic manifestations

Further management by GI/Colorectal surgery was recommended, along with annual surveillance and thyroid scanning. He subsequently underwent labwork, including a complete blood count and metabolic panel, which were reported unremarkable. He also underwent CT abdomen and pelvis with contrast, which was only remarkable for fatty infiltration along the falciform ligament, but no evidence of desmoid tumors, one of the extracolonic manifestations FAP (Figure 7).

Due to his diagnosis of FAP, we informed the patient of his higher risk of developing gastric and duodenal polyps. Hence, he was scheduled for an upper GI endoscopy in February 2020. The upper GI endoscopy showed a normal esophagus, a prominent antrum, and few duodenal polyps, which were tubular adenomas on histopathology. The patient had been advised to repeat upper GI endoscopy in one year for surveillance or present earlier if symptomatic.

During this entire period, the patient was established with our colorectal surgeons and informed who informed him about the importance of surgery in his case. Surgical options were offered, including total proctocolectomy with an ileal pouch or total abdominal colectomy with ileorectal anastomosis. The pros and cons of each surgery and the associated risks were discussed in detail with the patient. The patient successfully underwent a robotic-assisted total abdominal colectomy with ileorectal anastomosis in November 2020. The histopathology of the surgical specimen from the colon revealed multiple tubular adenomas (Figures 1-4).

His postoperative period was unremarkable, except for minor episodes of nausea and vomiting. Since then, the patient has had a follow-up appointment with his colorectal surgeon and gastroenterologist. He was advised to get a flexible sigmoidoscopy every six months and yearly upper GI endoscopy for surveillance. As per the most recent update about the patient, he underwent an upper GI endoscopy in July 2021, which showed normal esophagus, normal stomach, and few duodenal polyps, which were tubular adenomas on histopathology. He also underwent a flexible sigmoidoscopy in July 2021. A 3 mm polyp in the rectum and a 3 mm polyp in the anus were found, which were also found to be tubular adenomas on histopathology. Follow-up appointments with his specialists are currently scheduled for the patient.

## Discussion

Epidemiology

Familial Adenomatous Polyposis is a heritable syndrome with a frequency of one case for every 8000 to 100000 live births [1]. The disease affects both the sexes equally and has a constant frequency worldwide [1].

About 85% of the colorectal cancers are sporadic, and the rest of the 15% are considered familial. Out of the 15% familial colorectal cancers, familial adenomatous polyposis accounts for less than 1% of the cases [2].

Etiopathogenesis

FAP is a genetic disorder resulting from a mutation in the adenomatous polyposis coli (APC) gene [2]. Most FAP patients have a family history of colorectal polyps and cancer. However, 25% to 30% of them are "de novo," without clinical or genetic evidence of FAP in the family, which can be partially explained by germline mosaicism. Classic FAP is inherited as an autosomal dominant trait and results from a germline APC mutation [2]. A subset of individuals with clinical features of FAP will instead carry a mutation in the MUTYH ( mutY deoxyribonucleic acid glycosylase) gene [2].

Genetics

APC is a classical tumor suppressor gene located on the long arm of chromosome 5 in-band q21 (5q21). It encodes a large protein needed for transduction and transcription activities of the cell [3]. It also has a role in cell adhesion and cell migration [3]. The protein plays a significant role in Wnt, which are signaling pathways that are a group of signal transduction pathways that begin with proteins that pass signals into a cell through cell surface receptors. Hundreds of mutations of the APC gene have been recognized [3]. They have included insertions, deletions, nonsense, and missense mutations, resulting in a truncated or non-functional protein.

In the absence of the APC gene, as happens in these familial cancers, there is the absence of Wnt, beta-catenin is not degraded and leads to the production of target genes, including the c-Myc gene, which in turn leads to the production of a proto-oncogene [3].

The mutation's location is also associated with the age of onset, degree, the severity of the polyps, presence or absence of extracolonic manifestations, and even patient survival [4,5]. Mutations contributing to classical FAP occur between exon 5 and the 5' portion of exon 15. In contrast, those associated with the attenuated FAP tend to cluster in the extreme 5' portion of the gene and the 3' portion of exon 15 proximal to codon 1517 or distal to codon 1900 [6].

Mutations between codons at different locations have been associated with retinal lesions (congenital hypertrophy of the retinal pigment epithelium [CHRPE], desmoid tumors, duodenal polyposis, and development) of medulloblastoma [2].

Clinical Features

Two clinical variants of familial adenomatous polyposis (FAP) have been described.

(1) Classical FAP and (2) Attenuated FAP (AFAP), depending on where the mutation occurs in the APC gene [6]. 

(1) Classical FAP is characterized by the development of hundreds and thousands of polyps throughout the colon. By definition, it has to have a minimum of 100 polyps. The polyps develop in the patients by the second or third decade of life and typically by early teenage years.

If the condition is not detected or not treated, 100% of these patients will develop colorectal cancer between 35 and 43 years of age and with a mean of 39 years [7].

(2) Attenuated FAP (AFAP) is the less severe form of FAP and characteristically has about 30 polyps [8]. They have a later onset of the disease, and the chances of progression to colorectal cancer are about 70% [8].

Extracolonic manifestations: a plethora of extracolonic manifestations occur in FAP, including both benign and malignant lesions.

Gastrointestinal Tract:

(1) Duodenal adenomas are present in 30% to 70% of patients, and there is a very high lifetime risk of developing them [9]. These are most commonly found in the second and third portions of the duodenum. These assume great importance, as they have a 4% to 12% lifetime risk of becoming malignant and contribute as the second most common cause of death of these patients [10]. There is a 200 to 300 higher chance of having an ampullary tumor in patients with FAP than the general population, and they also have a higher risk of becoming malignant [11].

(2) Most patients with FAP have polyps in the gastric fundus or body of the stomach. These are small, sessile polyps with evidence of normal gastric corpus-type epithelium arranged in a disorganized manner or low-grade dysplasia, with rare progression to cancer [12]. 

(3) Intraabdominal desmoid tumors are found in about 15% of patients with FAP. Even though they do not metastasize, they can contribute to significant morbidity and mortality due to compression, obstruction, or encasement of adjacent structures [13].

(4) Hepatoblastomas, which are embryonal tumors, are more common in patients with FAP [14].

Head and Neck:

(1) Congenital hypertrophy of the retinal pigment epithelium (CHRPE) is an asymptomatic lesion. On the slit lamp, these appear as round to oval-shaped dark, discrete pigmented areas are seen in the ocular fundus [15]. The presence of multiple and bilateral CHRPE lesions is a clinical marker of the disease. 

(2) FAP patients have a higher risk of developing papillary thyroid cancer [16]*.*

 

Diagnosis 

If clinical suspicion arises, patients should be evaluated with a colonoscopy. Referral to a geneticist is needed for a definitive diagnosis. The presence of a germline mutation in the APC gene establishes the diagnosis of FAP. Most geneticists, however, test for both FAP- and MUTYH-associated polyps due to overlapping clinical presentation. Genetic testing should be offered to all first-degree relatives. The patients also need upper GI endoscopy to rule out adenomas in the upper gastrointestinal tract. They would also need abdominal and pelvic imaging through CT scan and MRI to rule out desmoid tumors. Eye examination, including referral to an ophthalmologist, is warranted in case of eye complaints. Lab work does not show evidence of any characteristic findings.

Differential diagnosis can include:

(a) Multiple lymphoid aggregates

(b) Peutz-Jeghers syndrome

(c) Lynch syndrome (hereditary non-polyposis colorectal cancer) 

(d) Multiple adenomatous polyps may also be found in cases with autosomal recessive MUTYH-associated polyposis (MAP) or Polymerase proofreading-associated polyposis (PPAP)

Genetic testing and histopathologic diagnosis can help in the differential diagnosis.

Recommendations for Genetic Counseling and Testing

The American Society for Gastrointestinal Endoscopy (ASGE) says that all patients with or suspected of having FAP undergo genetic counseling and testing [17]. The ASGE recommends testing for mutations of the adenomatous polyposis coli (APC) gene for confirming a diagnosis of FAP. The ASGE recommends APC gene testing and screening examinations for children starting at 10 and 12 years and starting at 18-20 years if attenuated FAP is suspected. The American College of Gastroenterology (ACG) recommends that genetic testing of patients with suspected adenomatous polyposis syndromes should include APC and MUTYH gene mutation analysis [14]. Genetic counseling is recommended for all first-degree relatives of patients with confirmed polyposis syndrome. All female relatives of the index case should receive pre-pregnancy counseling.

 

Recommendations for Endoscopy in FAP

Children suspected of having FAP should start getting screened at the age of 10 to 12 years; sigmoidoscopy is adequate for screening purposes. If no polyps are found on initial sigmoidoscopy, they should undergo follow-up screening every two years, starting in their late teen years. If, however, polyps are detected in the rectosigmoid colon, then the recommendations state that the child should undergo a complete colonoscopy to assess the degree and severity of polyposis and excise the larger polyps [17]. The ASGE recommends surveillance colonoscopy in patients confirmed to have FAP [17]. The ACG recommends that individuals at risk for or affected with the classic Adenomtosis polyposis (AP) syndromes undergo screening for colorectal cancer either by annual colonoscopy or flexible sigmoidoscopy starting at puberty [14].

Research is currently underway to use gene therapy to correct the underlying genetic error using liposomes as vectors. More research is needed to find the most effective gene therapy that could be useful in these cases.

Recommendations for Screening for Average Risk Population

Patient age is an important risk factor for colorectal cancer development, and almost 95% of new cases are seen above 45 years of age [18]. These incidence rates are even higher in the African-American, Native Alaskan, and American Indian populations [18]. In its most updated 2021 guidelines, the USPSTF now recommends colorectal cancer screening for asymptomatic average-risk patients to be started at 45 years [18]. 

Surgical Management 

The chances of development of colon cancer are almost 100%, and appropriately timed prophylactic colectomy is extremely important. The major surgical options available are prophylactic removal of the large intestine: colectomy with ileorectal anastomosis (IRA) and proctocolectomy with ileal pouch-anal anastomosis (IPAA). There are no guidelines regarding the timing of surgery [14,19]. Most patients with classical FAP are recommended surgery between 15 and 25 years of age [14,19].

Factors to consider in deciding the type of surgery include but are not limited to the patient's age, the rectal and colonic adenoma load, location of the APC mutation, wish for pregnancy, and risk of development desmoids [14,19].

IRA is considered a simpler and single staged procedure with a lower complication rate, though concerns for rectal cancer remain, and yearly proctoscopy is recommended [14,19]. On the other hand, IPAA is considered a more extensive procedure and includes pelvic dissection with associated complications and a higher risk of re-operation [14,19]. It is recommended that IPAA be preferable where adenoma load is high, or the chances of adenoma load are high, the risk of desmoid development is high, but might be avoided in those desiring pregnancy [14,19].

There was no significant difference between the procedures regarding sexual dysfunction, dietary restriction, or postoperative complications. The final decision lies with personal preference and the availability of surgical expertise [14,19].

## Conclusions

Family history is important in many disease processes due to the familial nature of the underlying pathology. Familial adenomatosis polyposis is one of them. Although rare, it can have devastating effects if left undiagnosed and untreated. This case reinforces the importance of checking proper history, including family history, by all the clinicians.
